# Elevated glucose represses lysosomal and mTOR-related genes in renal epithelial cells composed of progenitor CD133^+^ cells

**DOI:** 10.1371/journal.pone.0248241

**Published:** 2021-03-25

**Authors:** Swojani Shrestha, Sandeep Singhal, Donald A. Sens, Seema Somji, Bethany A. Davis, Rachel Guyer, Spencer Breen, Matthew Kalonick, Scott H. Garrett

**Affiliations:** 1 Department of Pathology, School of Medicine and Health Sciences, University of North Dakota, Grand Forks, North Dakota, United States of America; 2 Translational Genomics Research Institute, Phoenix, Arizona, United States of America; Center for Molecular Biotechnology, ITALY

## Abstract

Hyperglycemia is one of the major health concern in many parts of the world. One of the serious complications of high glucose levels is diabetic nephropathy. The preliminary microarray study performed on primary human renal tubular epithelial (hRTE) cells exposed to high glucose levels showed a significant downregulation of mTOR as well as its associated genes as well as lysosomal genes. Based on this preliminary data, the expression of various lysosomal genes as well as mTOR and its associated genes were analyzed in hRTE cells exposed to 5.5, 7.5, 11 and 16 mM glucose. The results validated the microarray analysis, which showed a significant decrease in the mRNA as well as protein expression of the selected genes as the concentration of glucose increased. Co-localization of lysosomal marker, LAMP1 with mTOR showed lower expression of mTOR as the glucose concentration increased, suggesting decrease in mTOR activity. Although the mechanism by which glucose affects the regulation of lysosomal genes is not well known, our results suggest that high levels of glucose may lead to decrease in mTOR expression causing the cells to enter an anabolic state with subsequent downregulation of lysosomal genes.

## Introduction

There is strong evidence that the renal proximal tubule is involved in the early stages, and subsequent progression, of diabetic kidney disease (DKD) [1–5]. These human studies, as well as studies utilizing animal models, have been complimented by the employment of *in vitro* cell culture models of putative proximal tubular cells to study a large range of factors associated with DKD. For humans, the most frequently used model is mortal cultures of renal tubular epithelial cells (RTE) isolated from the renal cortex of the human kidney, an HPV16 E_6_/E_7_ immortalized culture from the above cells identified as HK-2 cells, and a recently established immortalized culture of the above primary cultures using a construct containing hTERT called RPTEC/TERT1 [6]. All the three models express many of the features of proximal tubule differentiation, but they fail to express other important features such as a brush border on the apical surface, the SLC5A2 Na^+^-coupled glucose transporter, and an altered transepithelial resistance [7]. A recent examination of these 3 human cell culture models of the proximal tubule disclosed that the hRTE and RPTEC/TERT1 cells contained two cell populations, the majority co-expressing CD24 and CD133 and the remainder expressing CD24 but not CD133. In contrast, the number of HK-2 cells co-expressing CD133 and CD24 was below 20%. In a subsequent study, the RPTEC/TERT1 cells were sorted into two cell lines, one that expresses CD133 and CD24 and another one that expresses only CD24 [6]. The cells co-expressing CD133 and CD24 were shown to produce nephrospheres, formed branched tubule-like structures when grown on the surface Matrigel™, and were able to undergo neurogenic, adipogenic, and tubulogenic differentiation. The cells expressing only CD24 and not CD133 did not show these features. The features displayed for the CD133 and CD24 expressing cells are consistent with human renal progenitor cells that have been identified as those that are able to regenerate tubules following renal damage [8–12], with Bussolati et al. being the first to isolate these cells [8]. The CD24 and CD133 expressing cells in culture appear to represent the scattered progenitor cells localized to the proximal and distal tubules of the intact kidney and do not possess the ability to also differentiate into podocytes [10]. This is in contrast to those expressing CD106 along with CD133 and CD24 which can only differentiate into podocytes. The role of renal progenitors has focused on glomerular lesions with less emphasis on the regeneration of tubular components [10, 11]. These studies, taken together, suggest *in vitro* cultures, such as the hRTE and RPTEC/TERT1 with properties of the proximal tubule may represent a majority population of progenitor cells responsible for tubular regeneration in the human kidney.

This laboratory examined the effect of elevated glucose concentrations on the hRTE cells several years ago. These studies demonstrated that elevated glucose concentrations reduced paracellular transport as noted through a loss of dome formation (a measure of vectorial active transport), altered electrophysiological parameters, and ultrastructural analysis [13]. The hRTE cells were also shown to accumulate sorbitol when exposed to elevated glucose concentrations [14]. A decision was made not to pursue the studies further when it was demonstrated that phlorizin did not inhibit the short circuit current of hRTE cells grown on permeable supports, suggesting the absence of the sodium-coupled glucose transporter (now identified as SLC5A2) that localizes specifically to the proximal tubule [15]. With the new information that the hRTE cells have a majority population of cells co-expressing CD133 and CD24, a global gene expression analysis of the hRTE cells exposed to elevated concentrations of glucose was performed. The data obtained from this analysis is discussed in the present manuscript.

## Materials and methods

### Cell culture

Human renal tubular epithelial cells were obtained from cortical tissue of nephrectomies due to renal cell carcinoma. Tissue was obtained following accession and examination by surgical Pathology and, after completion of diagnostic protocols, informed consent was waved and was approved by the Medical University of South Carolina Institutional Review Board for Human Research [16]. All methods were performed in accordance with the relevant guideline and regulations with regard to the Declaration of Helsinki on ethical principles for medical research involving human subjects. Once the initial cultures reached confluency, each T-75 flask was frozen in liquid nitrogen in a single vial. In this study, these previously isolated hRTE stock cultures were used in experimental protocols. The cells were grown using serum-free conditions as previously described by this laboratory [16, 17]. The growth formulation consisted of a 1:1 mixture of Dulbecco’s modified Eagles’ medium and Ham’s F-12 growth medium supplemented with selenium (5 ng/ml), insulin (5 μg/ml), transferrin (5 μg/ml), hydrocortisone (36 ng/ml), triiodothyronine (4 pg/ml), and epidermal growth factor (10 ng/ml). The cells were fed fresh growth medium every 3 days, and at confluence, the cells were sub-cultured using trypsin–ethylenediaminetetraacetic acid (0.05%, 0.02%). For use in experimental protocols, cells were subcultured at a 1:2 ratio, allowed to reach confluence (7 days following subculture) and fed with 5.5, 7.5, 11 or 16 mM glucose for 7 days followed by sub-culturing the cells for 3 passages (P3) in media containing 5.5, 7.5, 11 or 16 mM glucose. Typically, the cells were passaged, 1:2 for three passages before the initiation of the experiment.

### Exposure of glucose treated hRTE cells to sorbinil

Human RTE cells were grown using serum-free conditions as previously described [16, 17]. At confluence, the cells were fed with 5.5, 7.5, 11 or 16 mM glucose or glucose and 100 μM sorbinil for 7 days, following which they were sub-cultured for 3 passages in their specific media and were harvested at confluency at P3 for further analysis.

### RNA isolation and real time quantitative polymerase chain reaction

Total RNA was isolated using Tri Reagent (Molecular Research Center, Inc., Cincinnati, OH) as described previously [18]. The measurement of CLCN7, NPC2, LIPA, RRAGD, SQSTM1, IGF2R, NEU1, mTOR, LAMP1, EIF4EBP1, RPTOR, RICTOR, FKBP11, FKBP2, DEPTOR, MLST8, RHEB and TCS1 mRNA expression was assessed with reverse transcription quantitative polymerase chain reaction (RT-qPCR) using commercially available primers (Bio-Rad Laboratories) as described previously [19, 20]. For analysis, 0.1 μg of total RNA was subjected to complimentary DNA (cDNA) synthesis using the iScript cDNA synthesis kit (Bio-Rad Laboratories) in a total volume of 20 μl. Real-time RT-qPCR was performed using the iTaq Universal SYBR Green Supermix (Bio-Rad Laboratories) with 2 μl cDNA and 0.2 μM primers in a total volume of 20 μl in a CFX96 real time detection system (Bio-Rad Laboratories). Amplification was monitored by SYBR Green fluorescence. Cycling parameters consisted of 40 cycles of denaturation at 95°C for 15 s, annealing at 60°C for 30 s, and extension at 72°C for 30 s, which gave optimal amplification efficiency. The resulting levels were normalized to the change in β-actin expression.

### Western analysis of lysosomal markers and mTOR associated genes

The expression of protein was determined by Western blotting using protocols that have been previously published by this laboratory [20]. Briefly, 20 μg of total cellular protein was separated by SDS–polyacrylamide gel electrophoresis using TGX AnyKd SDS polyacrylamide gel (Bio-Rad Laboratories) and transferred to a hybond-P Polyvinylidene difluoride membrane (Amersham Biosciences). Membranes were blocked in antibody dilution buffer (Tris-buffered saline containing 0.1% Tween-20 (TBS-T) and 5% (wt/vol) nonfat dry milk) for 1 h at room temperature. After blocking, the membranes were probed with the appropriate primary antibody in blocking buffer overnight at 4°C. The source of the antibodies along with their dilutions is listed in S9 Table. After washing 3 times in TBS-T, membranes were incubated with the appropriate anti-mouse or anti-rabbit antibody secondary antibody (1:2000) in antibody dilution buffer. The blots were visualized using the Phototope-HRP Western blot detection system (Cell Signaling Technology). Westerns were normalized to *β*-actin by stripping blots of previous antibodies and reprobing with the antibody for *β*-actin (ab8227) from Abcam Inc. (Cambridge, MA).

### Immunofluorescence/co-localization staining

As methodology reported before [6], cells were grown on coverslips and fixed with 3.7% formaldehyde for 15 min, followed by permeabilization using 0.1% Triton-X 100 for 10 min. The coverslips were washed 3 times using PBS for 5 min; blocked in 3% BSA for 30 min and incubated with primary antibody for 40 min at 37°C. Co-localization of mTOR and LAMP-1 was performed by mixing both primary antibodies and adding to the coverslips, and then incubated for 40 min at 37°C. The list of the primary antibodies used, along with their source, catalogue numbers and dilutions is provided in S9 Table. The coverslips were washed 3 times with PBS for 3 min each. The primary antibody was detected by incubating cells with species specific Alexa-Fluor 488 or Alexa-Fluor 568 secondary antibody for 30 min at 37 ^o^C. The coverslips were washed 3 times with PBS and mounted with Prolong Diamond Antifade Mountant with DAPI (Life Technologies). The stained cells were observed and imaged using Olympus FV3000 scanning confocal microscope. Two coverslips per sample were set up and z-stack images of a minimum of 5 fields per coverslips were captured with 40x objective lens. Co-localization analysis of z-stack images was performed using Olympus cellSens software.

### Statistical analysis

Statistical analysis consisted of one-way ANOVA with Dunnet’s multiple comparisons test using GraphPad PRISM 7 software. All experiments and exposures were done in triplicates and unless otherwise noted, all graphs are plotted as the mean ± SEM of triplicate determinations.

The R/Bioconductor package bead array [21] was applied to analyze the Illumina Bead Array data and convert into convenient R classes. The package also allows quality assessment on the raw data. The processed gene expression data contains 12 samples (triplicate of four glucose concentration) with expression of 47323 probes. A principle component analysis (PCA) and Pearson correlation were performed to test the distribution of data and relationship between gene expression values at different concentrations [22, 23]. One-way Analysis of Variance (ANOVA) [24] method applied to identify the significant probes across different concentrations followed by post-hoc [25] analyses in order to identify which two levels are different. The Benjamini–Hochberg method [26] at a specified FDR level of 5% was applied to identify the threshold level of significance. In order to analyze the functional relevance of the differentially expressed genes, we performed KEGG [27] pathway enrichment analyses using the Database for Annotation, Visualization and Integrated Discovery (DAVID) [28] version 6.8 online tool. The results were validated using publicly available functional genomics data repository Gene Expression Omnibus (GEO, link: https://www.ncbi.nlm.nih.gov/geo/). Graphpad PRISM 7 and R/Bioconductor version 3.5.1 were used for the entire statistical analysis.

## Results

### Microarray analysis

**Fig 1:**
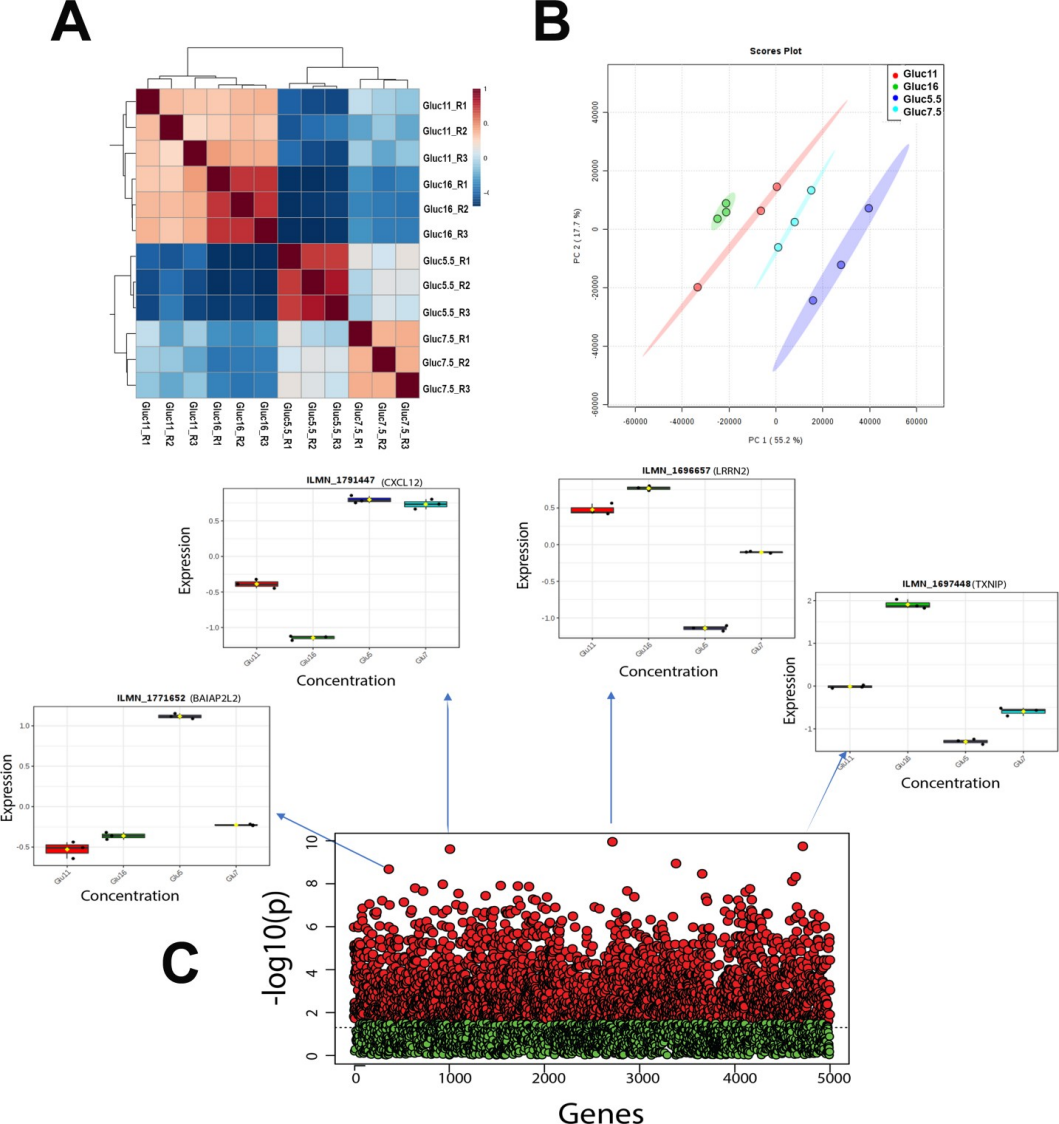
Cluster analysis. (A) Hierarchical cluster analysis and heat map of correlation; (B) Principal component analysis (PCA), between the replicates and different concentration using gene expression dataset. (C) ANOVA and post-hoc analysis test results. Red and green point represents the probes with below and above adjusted p-value (FDR) cutoff 0.05. X axis represents number of genes; Y axis represents negative adjusted p-value (FDR). Boxplots of top four probes (genes) are shown as an example to demonstrate the outcome variation, where X axis represents different concentrations; Y axis represents probe (gene) expression.

Microarray analysis was performed on total RNA isolated from hRTE cells cultured in the presence of 5.5, 7.5, 11.0 or 16.0 mM glucose for 3 serial passages. The cells were fed fresh growth media containing the above concentrations of glucose 24 hr prior to the isolation of the RNA. The summary of the data processing results is shown in S1 Table. Correlation analysis was performed to identify the strength of relationships between the replicates as well as the different concentrations with controls. The data (Fig 1A and 1B) showed that all replicates were clustered together and there was a very high anti-correlation between the controls and the highest concentration (correlation ranged: -0.694 to -0.635) which decreased gradually to medium (correlation ranged: -0.61 to -0.47) and further decreased with low concentration (correlation ranged: -0.09 to 0.13) (S2 Table). A total 2950 probes (S3 Table) corresponding to 2615 genes with FDR value below the threshold were selected for downstream pathway analysis. Fig 1C shows the statistical distribution of all probes. For any given gene list, the Database for Annotation, Visualization and Integrated Discovery (DAVID) identified a total of 52 (42 with p<0.05) KEGG pathways (S4 Table). To further investigate the biological context, the entire significant gene list was further divided into the up-regulated and down-regulated genes using fold-change (FC) values between control and the highest concentration. We found 1227 probes (corresponding to 1107 genes) were down-regulated (S5 Table) and 1723 probes (corresponding to 1536 genes) were up-regulated (S6 Table). To find the functional relevance, we again used DAVID tools for both the gene-lists to test the pathway association independently. The up regulated gene-list associated with a total of 42 (37 with p<0.05) KEGG pathways (S7 Table), whereas, the down-regulated gene-list associated with 30 KEGG (21 with p<0.05) (S8 Table) pathways. The four main pathways, 1) Protein processing in endoplasmic reticulum, 2) Lysosome, 3) Biosynthesis of antibiotics, and 4) Steroid biosynthesis were highly associated after FDR correction with a cutoff of 0.05.

**Fig 2:**
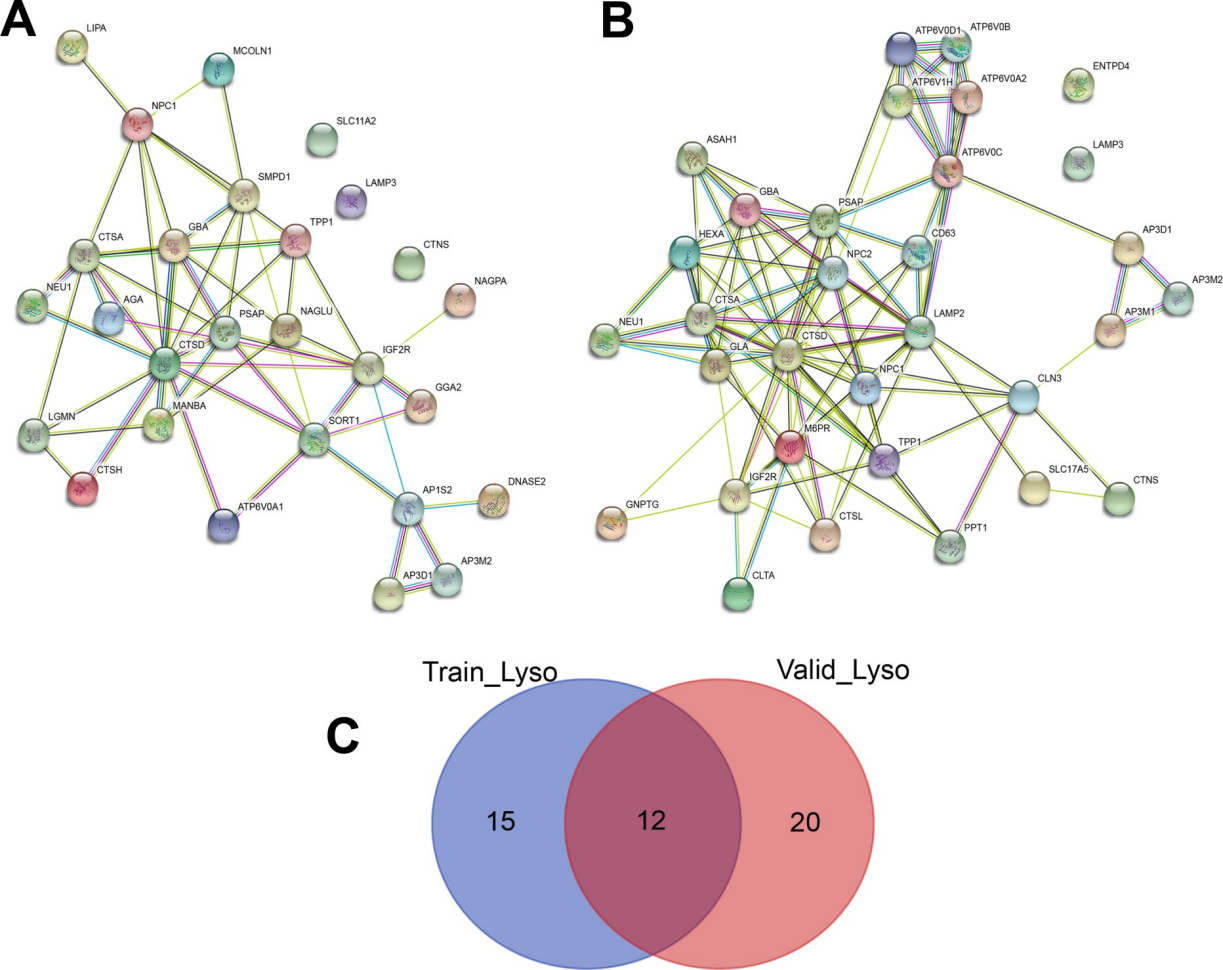
Gene-gene interaction network of lysosome pathways related genes. (A) training data and (B) validation data. The color saturation of the edges represents the confidence score of a functional association. (C) Venn diagram of Lysosome pathways related genes in training and validation data.

The program STRING imports data from experimentally derived protein–protein interactions through literature curation based on an analysis of known, direct or functional defined gene-gene interactions. Using this program, the linkages of lysosome genes of training data assembled into two distinct networks 1) centered by CTSD gene highly connected with NEU1, CTSA, GBA, PSAP, MNBA, IGF2R, and TPP1, and 2) subunits of AP3 adaptor-like complex such as AP3D1, and AP3M2 (Fig 2A). To validate the robustness of regulation network, lysosome genes of validation data were also tested using STRING. The result shows three distinct networks (Fig 2B). Interestingly, there were only 12 genes common between the training and validation lysosome gene list but both of the training gene-sets networks found were very similar in the validation gene-sets network (Fig 2C).

### Effect of high glucose on the expression of lysosomal genes

**Fig 3:**
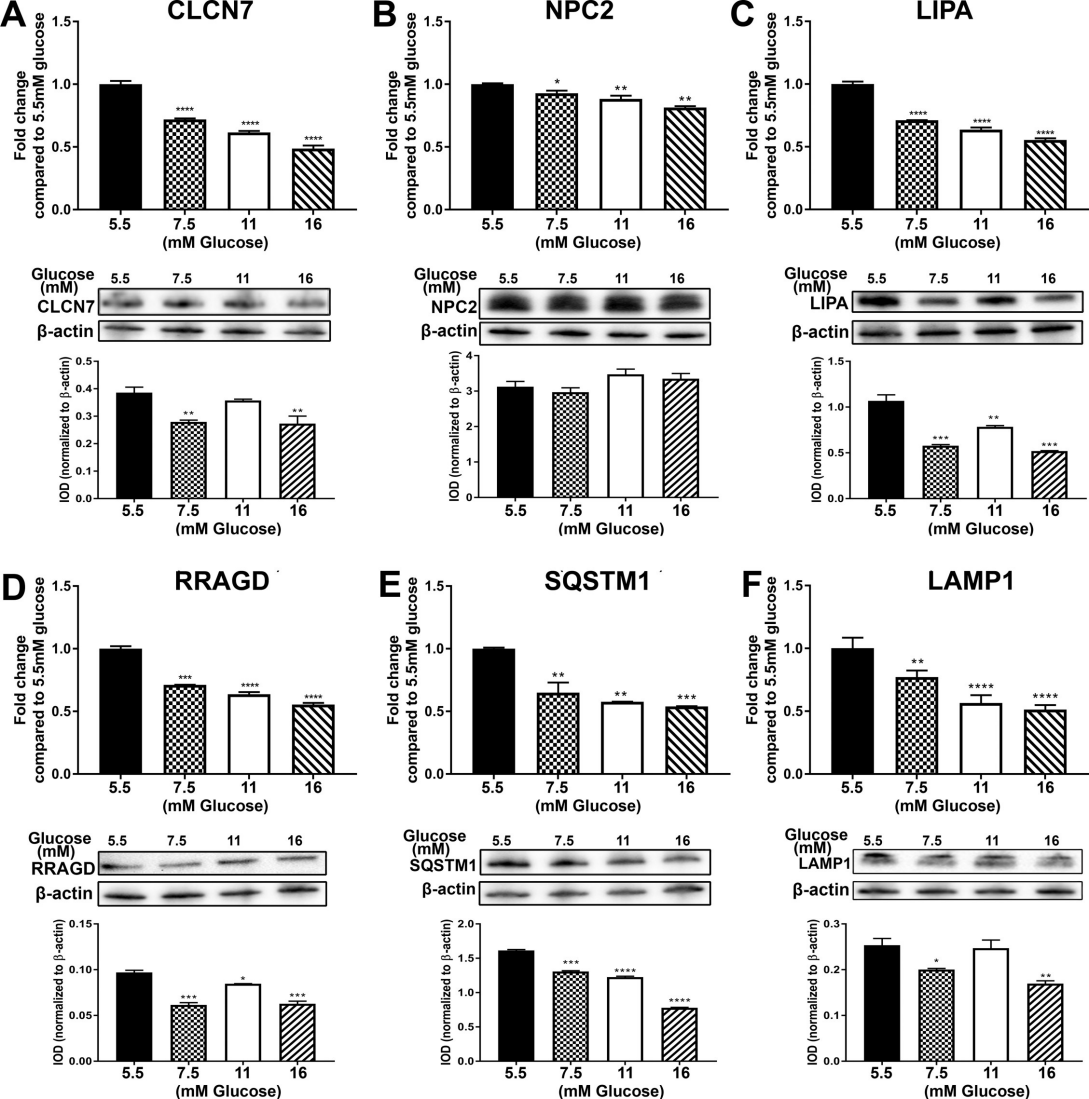
Expression of lysosomal markers in cultures of human proximal tubule cells treated with 5.5 mM, 7.5 mM, 11 mM and 16 mM glucose for 3 passages. RT-qPCR and Western blot analysis of (A) CLCN7; (B) NPC2; (C) LIPA; (D) RRAGD; (E) SQSTM1; (F) LAMP1. The top graphs in the figure represents PCR data whereas the bottom graphs represent the IOD of the Western blot analysis. ****; ***; **; * indicates significant differences in gene expression level compared to the control 5.5 mM glucose concentration at p-value of ≤ 0.0001; ≤ 0.001; ≤ 0.01; ≤ 0.05 respectively.

The above analysis of global gene expression identified the lysosome as the organelle most affected by elevated glucose concentrations. An analysis was undertaken to identify those genes from the global array that were associated with the lysosome and related pathways using the National Center for Biotechnology Information (NCBI), Gene Cards [29], DAVID [30, 31] and Reactome [32]. The results of this analysis identified 35 genes associated with the lysosome (Table 1). All the 35 genes were shown to be reduced in expression when exposed to 7.5, 11.0 and 16 mM glucose after 3 serial passages when compared to the 5.5 mM glucose control. Ten of these genes were further analyzed using total RNA obtained from cells grown on the 4 glucose concentrations at passage 1 (P1), passage 2 (P2) and passage 3 (P3). The results for the PCR analysis showed that the expression of these genes was similar to that found by global gene expression analysis (S1 Fig). There was a decrease in expression of all 10 of the genes. Initially, the expression of IGF2R, LGMN, MCOLN1, NEU1, LIPA, NPC2, SQSTM1 and RRAGD increased at P1 or P2, but by the third passage, the expression levels decreased significantly (S1 Fig). The expression of the other 2 genes CTSA and CLCN7 either did not change till P3 or it decreased and remained low for the rest of the study. The protein levels for 5 of the expressed genes as well as another lysosomal gene LAMP1 which was not altered in the global gene analysis was measured along with the mRNA at P3 and the data is presented in Fig 3A–3F. The expression of LAMP1 along with the other lysosomal genes showed a decrease in expression at P3 (Fig 3F). Thus our results indicate that initial exposure to elevated glucose has little effect on lysosomal gene expression, but longer exposure results in a marked reduction in the expression of these genes.

**Table 1:** Lysosomal genes identified by global gene expression.

Gene Symbol	Gene Name	Global Gene Expression	
		Induced (I)	Repressed (R) Fold Change at 16 mM
CLN3	battenin		R (0.67)
FYVE	coiled-coil domain containing (FYCO1)		R (0.76)
IFI30	lysosomal thiol reductase		R (0.68)
NAGLU	N-acetyl-alpha-glucosaminidase		R (0.67)
NPC1	intracellular cholesterol transporter 1		R (0.83)
NPC2	intracellular cholesterol transporter 2		R (0.77)
RRAGC	Ras related GTP binding C		R (0.49)
RRAGD	Ras related GTP binding D		R (0.35)
VPS11	CORVET/HOPS core subunit		R (0.74)
VPS39	HOPS complex subunit		R (0.77)
VPS41	HOPS complex subunit		R (0.61)
WDR48	WD repeat domain 48		R (0.75)
CTSA	cathepsin A		R (0.65)
CTSH	cathepsin H		R (0.58)
CLCN7	chloride voltage-gated channel 7		R (0.56)
CTNS	cystinosin, lysosomal cystine transporter		R (0.48)
DPP7	dipeptidyl peptidase 7		R (0.77)
FNBP1	formin binding protein 1		R (0.65)
HEXB	hexosaminidase subunit beta		R (0.74)
IGF2R	insulin like growth factor 2 receptor		R (0.44)
LGMN	legumain		R (0.54)
LIPA	lipase A, lysosomal acid type		R (0.50)
LDLR	low density lipoprotein receptor		R (0.51)
MANBA	mannosidase beta		R (0.66)
MCOLN1	mucolipin 1		R (0.46)
NEU1	neuraminidase 1		R (0.52)
OSTM1	osteopetrosis associated transmembrane protein 1		R (0.65)
PNPLA7	patatin like phospholipase domain containing 7		R (0.81)
PCSK9	proprotein convertase subtilisin/kexin type 9		R (0.53)
PSAP	prosaposin		R (0.67)
SQSTM1	sequestosome 1		R (0.57)
SLC29A3	solute carrier family 29 member 3		R (0.74)
SMPD1	sphingomyelin phosphodiesterase 1		R (0.75)
TPP1	tripeptidyl peptidase 1		R (0.43)
LAMP1	Lysosomal associated membrane protein 1		R (0.92)

### Effect of high glucose on the expression of mTOR associated genes

**Fig 4:**
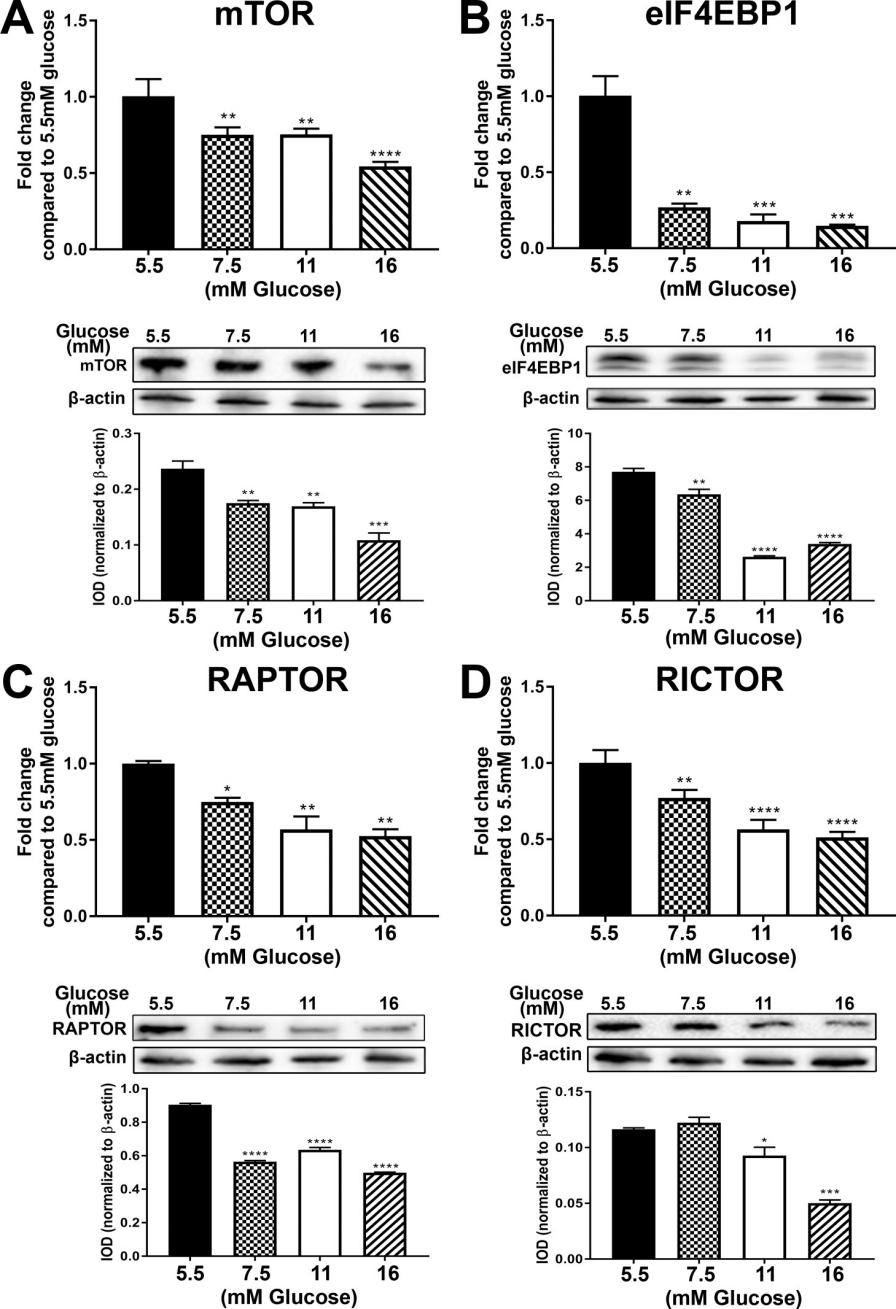
Expression of mTOR and associated genes. Cultures of hRTE cells were treated with 5.5 mM, 7.5 mM, 11 mM and 16 mM glucose for 3 passages. RT-qPCR and Western blot analysis of (A) mTOR; (B) EIF4EBP1; (C) RAPTOR; (D) RICTOR. The top graphs in the figure represent the PCR data whereas the bottom graphs represent the IOD of the Western blot analysis. ****; ***; **; * indicates significant differences in gene expression level compared to the control 5.5mM glucose concentration at p-value of ≤ 0.0001; ≤ 0.001; ≤ 0.01; ≤ 0.05 respectively.

Several of the above lysosomal genes were noted to have an association with mTOR and this led to an examination of this pathway. Table 2 lists the identified genes from the global gene analysis that were associated with mTOR and altered by elevated glucose concentrations, including the mTOR gene itself. Thirteen of the 15 genes were repressed and 2 of the genes had induced expression. The mRNA and protein levels of 4 of the genes was further analyzed in the P3 samples (Fig 4A–4D). There was a decrease in expression of all of the 4 genes (mTOR, eIF4EBP1, RAPTOR and RICTOR). The expression levels of the genes at all 3 passages is shown in S2 Fig. In addition, the expression of an additional gene LAMP1, which did not show any change in expression in the global gene analysis was also determined. For the genes mTOR, LAMP1, eIF4EBP1, RICTOR, DEPTOR, FKBP2, TSC1 and RHEB, there was an increase in expression at either P1 or P2, followed by a decrease in expression at P3. For the other genes RAPTOR, FKBP11 and MLST8, there was either no change in expression levels at P1 or P2 followed by a decrease at P3, or there was a decrease in expression at P1 and/or P2, followed by a decrease in P3 as well. Thus our data indicates that exposure to high levels of glucose can decrease the expression of mTOR and its associated genes.

**Table 2:** mTOR and associated genes identified by global gene expression.

Gene Symbol	Gene Name	Global Gene Expression	
		Induced (I)	Repressed (R)
FRAP1	mammalian target of rapamycin (mTOR)		R (0.66)
FKBP2	FKBP prolyl isomerase 2	I (1.24, 16mM)	R (0.95, 7.5 mM)
FKBP11	FKBP prolyl isomerase 11		R (0.47)
TSC1	TSC complex subunit 1		R (0.73)
EIF4EBP1	eukaryotic translation initiation factor 4E binding protein 1		R (0.67)
FNIP1	folliculin interacting protein 1		R (0.67)
OGT	O-linked N-acetylglucosamine transferase 2		R (0.55)
SKP2	S-phase kinase-associated protein 2	I (1.22, 16 mM)	
RNF152	E3 ubiquitin-protein ligase		R (0.85)
CREB3L2	cyclic AMP-responsive element-binding protein 3-like protein 2		R (0.66)
RAPTOR	regulatory associated protein of MTOR complex 1		R (0.76)
RICTOR	independent companion of MTOR complex 2		R (0.71)
DEPTOR	domain containing MTOR interacting protein		R (0.69)
MLST8	MTOR associated protein		R (0.75)
RHEB	Ras homolog, MTORC1 binding		R (0.80)

### Effect of elevated glucose exposure on the intracellular localization of lysosomal proteins

**Fig 5:**
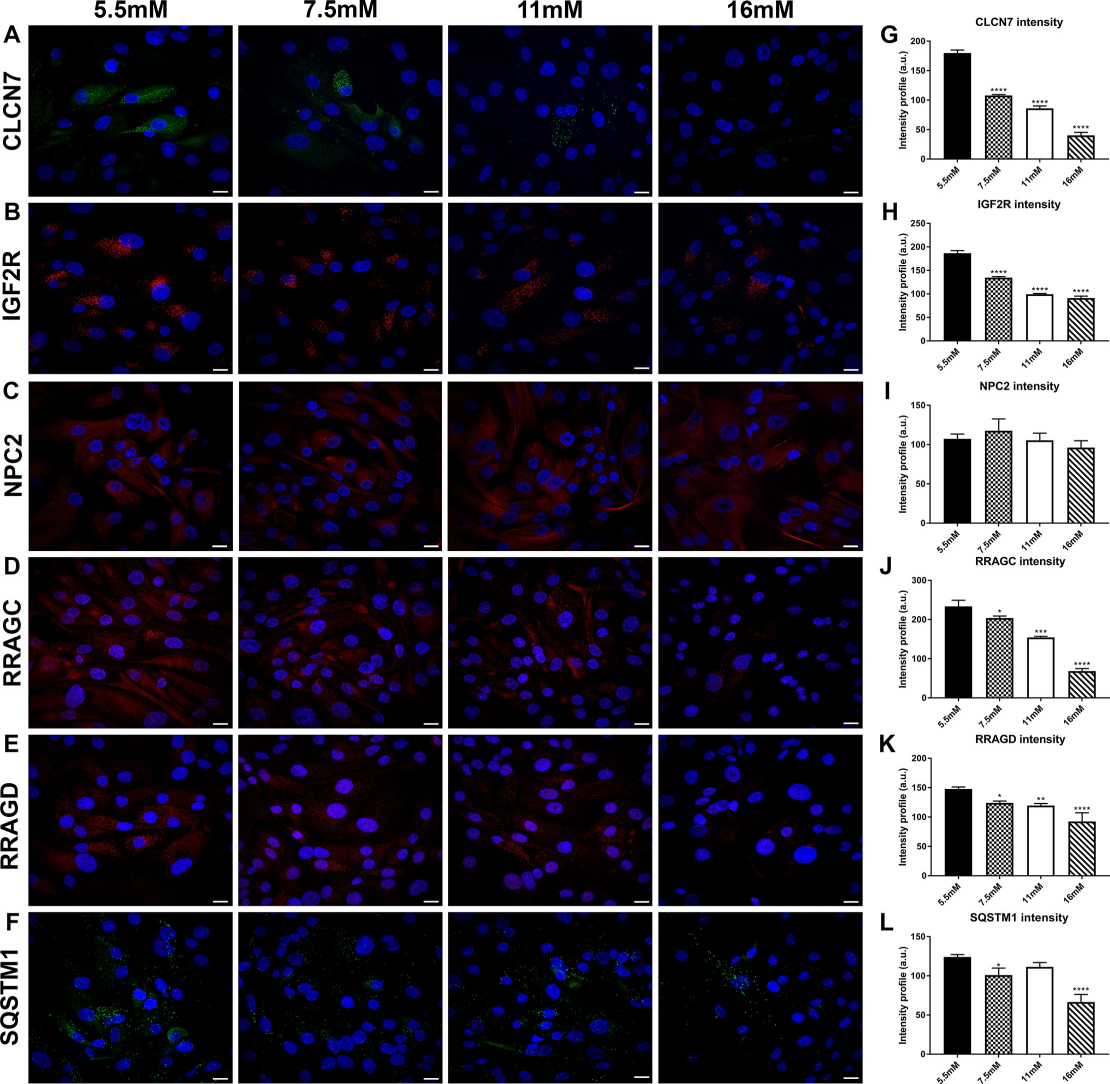
Immunofluorescence staining of lysosomal markers in glucose treated human renal tubule cells cultured *in vitro*. Staining for (A) CLCN7; (B) IGF2R; (C) NPC2; (D) RRAGC; (E) RRAGD; (F) SQSTM1 in hRTE cells treated with 5.5 mM, 7.5 mM, 11 mM and 16 mM glucose concentrations and harvested at passage three. Each protein is stained in green or red color and DAPI is stained in blue. (G-L) Intensity profile of images of CLCN7; IGF2R; NPC2; RRAGC; RRAGD; SQSTM1 performed by Olympus cellSens software. ****; ***; **; * indicates significant differences in gene expression level compared to the control 5.5mM glucose concentration at p-value of ≤ 0.0001; ≤ 0.001; ≤ 0.01; ≤ 0.05 respectively. Scale bar = 21.16 μm. Magnification x400.

Confluent cultures of hRTE cells grown on 5.5, 7.5, 11, 16 mM glucose were placed on coverslips after the third serial passage and utilized for immunofluorescence staining of lysosomal proteins. Six proteins CLCN7, IGF2R, RRAGC, RRAGD, NPC2 and SQSTM1 were examined and all showed cytoplasmic punctate staining consistent with a lysosomal localization (Fig 5A–5F). The intensity of staining for all the assessed proteins, except NPC2, was distinctly lower in the cells treated with 11 and 16 mM glucose compared to the 5.5 mM glucose control (Fig 5G–5L). The intensity profile was measured using cellSens image analysis software for each of the confocal images.

**Fig 6:**
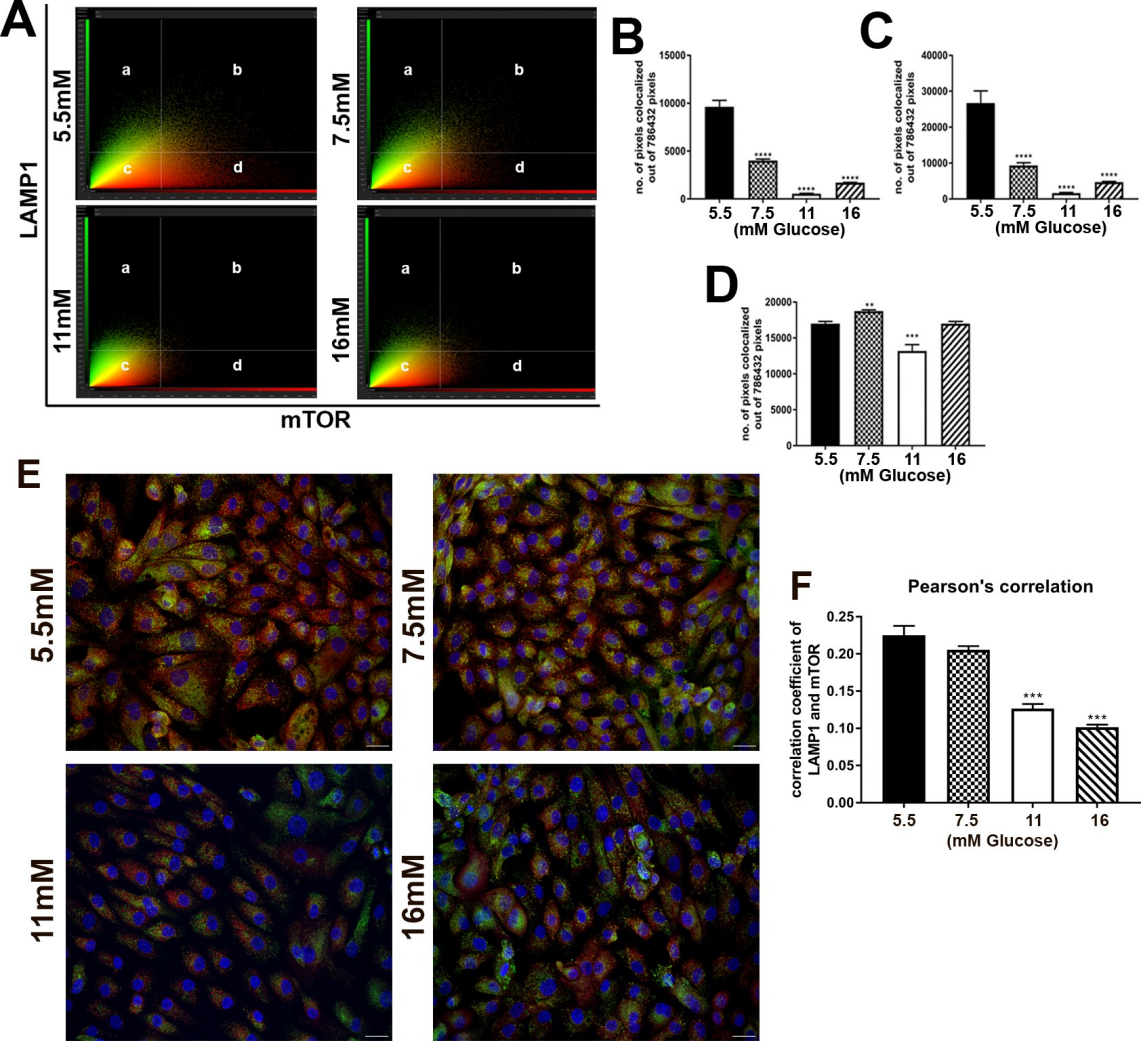
Colocalization of mTOR and LAMP1 in hRTE cell cultures. Human RTE cells were treated with 5.5 mM, 7.5 mM, 11 mM and 16 mM glucose concentrations for 3 passages. (A) Scatterplot analysis of immunocolocalized mTOR (red) and LAMP1 (green) proteins in which (a) represents pixels that determine LAMP1 staining; (b) represents colocalized mTOR as well as LAMP1 pixels; (d) represents pixels that determine mTOR staining; (c) represents pixels below the threshold. (B) Graphical representation of number of immunocolocalized mTOR and LAMP1 pixels. (C) Graphical representation of number of pixels that determines mTOR staining only. (D) Graphical representation of number of pixels that determines LAMP1 staining only. (E) Double stained immunofluorescence images for mTOR (red) and LAMP1 (green). Nuclei are stained with DAPI (blue). (F) Colocalization based on Pearson’s correlation. ****; ***; ** indicates significant differences in gene expression level compared to the control 5.5mM glucose concentration at p-value of ≤ 0.0001; ≤ 0.001; ≤ 0.01 respectively. Scale bar = 21.16 μm. Magnification x400.

Co-localization of mTOR protein with LAMP1 protein was determined in the cells treated with 5.5, 7.5, 11, 16 mM glucose after 3 passages in culture. The analysis is represented as a scatterplot, in which the threshold was determined based on no (zero) co-localized pixels out of the total pixels visualized in the unstained control cells (Fig 6A). The x-axis and y-axis represents the number of pixels for mTOR and LAMP1 respectively. The results showed that the % of co-localization decreased in the cells treated with 7.5, 11 and 16 mM glucose compared to the 5.5 mM control (Fig 6B). The channel representing mTOR (Fig 6C) showed a greater decrease in fluorescence intensity when compared to LAMP1 channel. (Fig 6D). The threshold calculations generated the double stained immunofluorescence images which identifies immunolocalized mTOR with LAMP1 (Fig 6E). Fig 6F shows the Pearson’s correlation of mTOR and LAMP1 colocalization, which also decreased. The number of pixels for single channels were also analyzed, representing the area of staining for each protein as the concentration of glucose increased. There was a large decreased in mTOR staining area (Fig 6C), but the area of staining for LAMP1 (Fig 6D) did not decrease at 16 mM and a small decrease at 11 mM, despite the prominent decrease in overall expression at the mRNA and protein levels (Fig 3F). Since LAMP1 is often used as a lysosomal marker this is suggestive of the number of lysosomes or the total membrane area of lysosomes did not change with glucose exposure.

### Expression of Na^+^-dependent glucose transporters in hRTE cells

**Fig 7:**
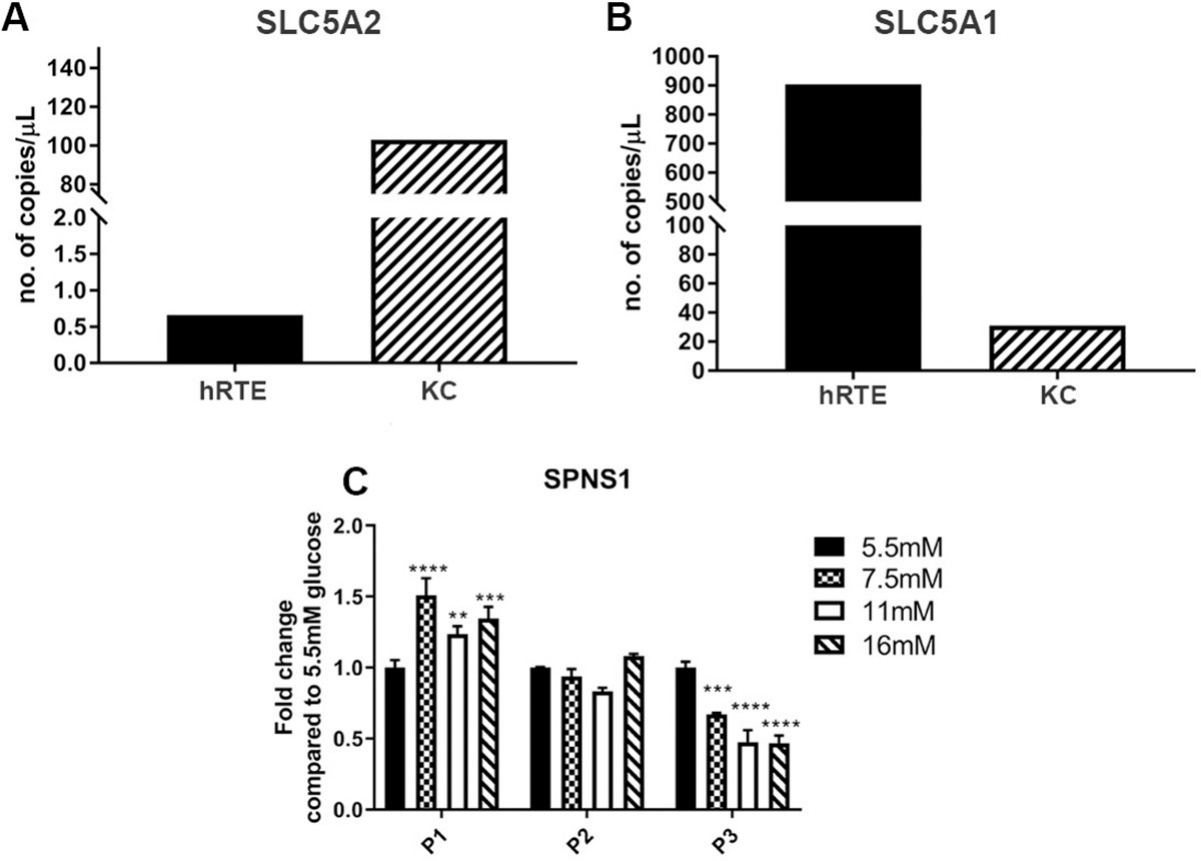
Expression of Na^+^- glucose transporters and sphingolipid transporter in cultures of hRTE cells and human kidney cortex tissue (KC). Expression of (A) SLC5A2 and (B) SLC5A1 in hRTE cells and kidney cortex (KC) total RNA sample. PCR analysis sample concentrations are plotted as copies per μl. (C) Expression of sphingolipid transporter 1 (SPNS1) in the hRTE cultures treated with 5.5 mM, 7.5 mM, 11 mM and 16 mM glucose concentrations at passage one, two and three. ****; ***; ** indicates significant differences in gene expression level compared to the control 5.5 mM glucose concentration at p-value of ≤ 0.0001; ≤ 0.001; ≤ 0.01 respectively.

A distinguishing feature of the proximal tubule is the presence on a low-affinity, high capacity Na^+^-glucose co-transporter in the brush border on the apical surface of the cell. This transporter, identified as SLC5A2, is mainly responsible for the reabsorption of glucose by the proximal tubule. The expression of SLC5A2 mRNA was determined on total RNA from the hRTE cells and it was found to be minimally expressed (Fig 7A). Total RNA from human kidney cortex was used as a control. In contrast, the expression of mRNA for the SLC5A1 transporter was shown to be highly expressed in the hRTE cells and to have marginal expression in the human kidney cortex (Fig 7B). The expression of mRNA for the Spinster gene (SPNS1) was also determined in the hRTE cells since this gene has been implicated in the lysosomal response to glucose [33]. It was found that the expression of SPNS1 decreased as the concentration of glucose exposure increased (Fig 7C).

### Expression of lysosomal genes following inhibition of sorbitol accumulation in hRTE cells exposed to elevated glucose

**Fig 8:**
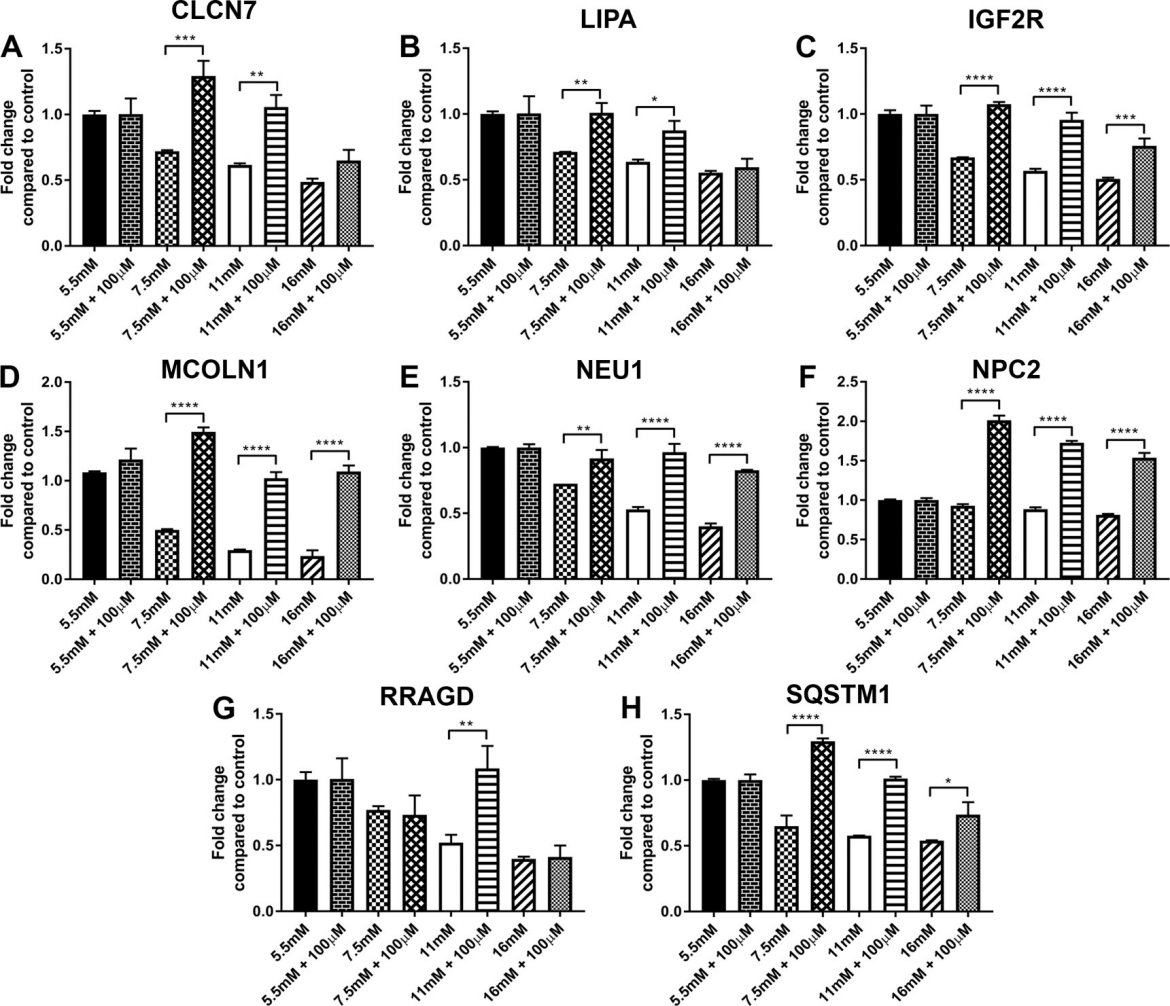
Expression of lysosomal markers in cultures of human proximal tubule cells. Human RTE cells were treated with 5.5mM, 7.5mM, 11mM and 16mM glucose with or without 100μM sorbinil for three serial passages. RT- qPCR analysis of (A) CLCN7; (B) LIPA; (C) IGF2R; (D) MCOLN1; (E) NEU1; (F) NPC2; (G) RRAGD and (H) SQSTM1. ****; ***; **; * indicates significant differences in gene expression level between hRTE cells treated with glucose only and glucose with sorbinil at p-value of ≤ 0.0001; ≤ 0.001; ≤ 0.01; ≤ 0.05 respectively.

Previously it has been shown that the hRTE cells can increase sorbitol as a function of elevated glucose concentration and levels of sorbitol can be reduced to near normal by treating the cells with the aldose reductase inhibitor, sorbinil [14]. The expression of eight lysosomal genes (CLCN7, LIPA, IGF2R, MCOLN1, NEU1, NPC2, RRAGD, and SQSTM1) was determined after treating the cells with 5.5, 7.5, 11 and 16 mM glucose and 100μM sorbinil (Fig 8A–8H). The results demonstrated that inhibition of sorbitol accumulation induced the expression of CLCN7 and LIPA at 7.5 and 11 mM glucose, but had no effect on the expression of these 2 genes at the highest dose of 16 mM glucose. The expression of IGF2R, MCOLN1, NEU1, NPC2 and SQSTM1 was induced at all 3 concentrations of glucose whereas the expression of RRAGD was only induced at 11 mM glucose.

## Discussion

The present study shows that elevated glucose concentrations can reduce the expression of genes associated with the lysosome and the related mTOR pathway in human cultured renal epithelial cells. The hRTE cell culture model was chosen for the present studies since they have recently been shown to be composed of approximately 60–70% of cells that possess both CD133 and CD24, with the remaining cells expressing CD24 and no CD133 [6, 7]. This is important, since as detailed in the introduction, *in vivo* studies have shown that progenitor cells capable of tubular regeneration always co-express CD133 and CD24. This has allowed this laboratory to suggest that hRTE cells, identified as possessing proximal tubule differentiation, are in fact composed mostly of progenitor cells. In the present study, this was further reinforced by the finding that the cells do not express the SLC5A2 glucose transporter that is responsible for the majority of glucose reabsorption in the kidney [34]. The hRTE cells were shown to express the SLC5A1 sodium coupled glucose transporter located downstream from the SLC5A2 transporter in the late portion of the proximal tubule. In the kidney, this transporter is only responsible for reabsorbing 3% of filtered glucose [35]. Our study demonstrated that the expression of SLC5A2 in the human kidney cortex is much higher than the expression in cultured hRTE cells. These findings allowed the laboratory to determine the effects of elevated glucose concentrations on a putative population of renal progenitor cells central to repairing tubular structures lost due to diabetic nephrotoxicity. The hRTE cells were chosen for examination even though they possessed a minority population of cells expressing CD24 and not CD133. One reason for this was the hRTE cells are not immortalized, but the primary culture can be serially passaged for 20 generations (when the split ration is 1–2) with constant morphology before experiencing a marked reduction in cell growth. This allows studies to be performed *in vitro* without the multitude of changes induced by immortalization. A second reason was that independent cultures of hRTE cells from different donors all contained an approximate ratio of 2:1 CD133/CD24 expressing cells and cells expressing only CD24 [6]. This suggested that there might be an important association between these two cell linages that contribute to their progenitor status. This is a possibility that might be missed in *in vivo* progenitor studies since CD24 alone is a frequent marker for a variety of tubules. The ratio of CD133/CD24 to CD24 alone in the present study did not change as a function of glucose concentration. Thus, the present study gives initial information on the effect of elevated glucose on the gene expression of putative renal tubule progenitor cells.

While there is a wealth of information on the post-transcriptional regulation of the protein components of both the mTOR complex and the lysosome, there is far less literature on the transcriptional regulation of mTOR and lysosomal gene expression [36–39]. This also appears to be especially true for the effect that glucose concentration might have for gene expression at both the transcriptional and translational level for the lysosomal and mTOR pathways. The only report the authors could find linking elevated glucose concentrations and gene expression to the lysosome was a report on the effects of elevated glucose on the metastatic potential of tongue squamous cell carcinoma [40]. While the manuscript itself focused on the regulation of glycolytic enzymes as a function of elevated glucose concentrations, their submission of microarray data to GEO, when examined by this laboratory, showed a relationship between elevated glucose and the lysosome. In addition, comparing the present array data with the data from the GEO submission showed a strong relationship when examined for gene-gene interactions. The only other report to link a variation in glucose concentration to post-transcriptional lysosomal events was the observation that changes in glucose concentration induces lysosome formation and drug sequestration [41]. Thus, our results which show that elevated glucose can down-regulate the expression of a large number of lysosomal genes and components of the mTOR complexes, including mTOR itself, appears to be a rather novel observation.

A clear mechanism for the down-regulation of lysosomal gene transcription was not evident from the present study. However, any mechanism to explain the down-regulation of these numerous genes will likely involve the transcription factor EB (TFEB). This view is derived from studies which showed that entire classes of lysosomal genes, including lysosomal hydrolases, lysosomal membrane permeases, and lysosomal-associated proteins are under coordinated transcriptional control of EB [38, 42, 43]. These studies showed that an E box-related consensus sequence was present in the promoter regions of many lysosomal genes. This consensus sequence was named CLEAR (coordinated lysosomal expression and regulated element). The CLEAR element is the target of a family of basic helix-loop-helix transcription factors known as the MiT/TFE proteins whose members include TFEB, TFEC, TFE3 and MITF. The transcription factor EB binds directly to CLEAR elements and stimulates the expression of their downstream target genes. In addition, the overexpression of TFEB induces a large expansion of the lysosomal compartment, when assessed by size, number and protein content [43]. TFEB also drives the expression of a number of proteins involved in autophagosomal initiation, elongation, substrate capture, and autophagosomal trafficking and fusion with lysosomes [38, 42]. The research of Palmieri and coworkers [42] identified direct targets of TFEB with a known role in lysosomal function. This included 26 genes classified under lysosomal hydrolases, 9 genes with lysosomal membrane, 10 genes as non-lysosomal genes involve in lysosome biogenesis, and 17 genes associated with autophagy. In the present study, elevated glucose resulted in a marked reduction in expression of 8 of the 26 genes classified as hydrolases, 5 of the 9 genes classified as lysosomal membrane associated, 5 of the 10 genes involved in lysosomal biogenesis, and 3 of the 17 genes involved in autophagy. The present study also identified ATP6VIC1 as the gene involved in lysosomal acidification. Overall, these results strongly suggest that TFEB, or an EB-interactive protein, influences the down-regulation of lysosomal related genes when the hRTE cells are exposed to elevated glucose concentrations.

Similar to the down-regulation of the lysosomal genes by elevated glucose concentrations, mTOR, and its associated genes were also down-regulated when hRTE cells were exposed to elevated glucose concentrations. The lysosome plays an essential role in sensing and signaling cellular nutrient status by recruiting mTORC1 to the lysosomal membrane where it acts as a master regulator of autophagy through sensing the level of amino acids. The post-translational regulation of mTOR and its binding partners have been reviewed extensively in recent years [36, 37, 44]. The only link between mTOR, the lysosome, and glucose identified at the post-translational level by the authors was a report that a permeably channel is located on the lysosomal surface that mediates mTORC1 activation by glucose [45]. This mechanism may involve SPNS1, a putative lysosomal sugar transporter, which controls mTOR activity [33]. The present study demonstrated that SPNS1 was down-regulated with elevated glucose concentration. The transcription factor Nrf2 has been shown to regulate the expression of mTOR through the recognition and binding to antioxidant response elements (AREs) in the promoter region of the gene [46]. The over expression of Nrf2 in a variety of cells lines resulted in the increase expression of the mTOR gene and also showed a relationship with the PI3K signaling pathway. Nrf2 activators have been proposed as possible therapeutic agents to diminish the nephropathy associated with diabetes [47] by up regulating the expression of phase II detoxifying enzymes. However, the microarray analysis in the present study did not identify a cellular stress response associated with the elevated glucose concentrations, in line with the finding of repression of mTOR gene expression and not an increase in expression.

Several of the lysosomal and mTOR related proteins that were down-regulated by elevated glucose were examined using confocal microscopy. The single fluorescent analysis of the CLCN7, IGF2R, RRAGC, RRAGD, NPC2 and SQSTM1 proteins all showed a lysosomal localization and a reduction in fluorescent intensity that correlated to the increased glucose concentrations. The results of this examination also showed that the number of lysosomes did not change significantly as a result of increasing glucose concentration. The single channel analysis of the LAMP1 protein also showed that the intensity of staining was decreased with increasing glucose concentration, but that the staining area (pixel number), indicative of lysosomal number or lysosomal membrane area were relatively unchanged due to the elevated glucose concentrations. Co-localization of mTOR with LAMP1 was decreased with increasing glucose concentrations indicating the dissociation of mTOR from the lysosomal membrane. The degree of inactivation of mTOR was difficult to determine since the amount of the mTOR protein itself was reduced when the cells were exposed to elevated glucose concentrations; however, the loss from the lysosomal membrane appeared to exceed the loss of mTOR protein. The dissociation of mTOR from the lysosomal membrane is associated mostly with amino acid starvation. However, in the present study cells were fed with fresh growth media the evening before mRNA and protein isolation, thus nutrient deprivation would not be a factor.

The reduction in the expression of the lysosomal and mTOR associated genes did not occur in full until the 3^rd^ passage of the cells on elevated glucose concentrations. Very few of these genes were reduced on the initial passage of the cells on high glucose, with a few more showing a reduction on the 2^nd^ passage. By the end of the 3^rd^ passage the cells would have been exposed to the elevated glucose concentrations for 30 days. Previous studies by the laboratory has shown that exposure of the hRTE cells to elevated glucose concentrations results in the accumulation of sorbitol [14]. This accumulation is rapid, occurring within 24 hr of exposure to elevated glucose concentration with accumulation being reduced by 70% in the presence of the aldose reductase inhibitor, sorbinil. The effect of sorbinil inhibition was tested on eight genes after 3 passages on elevated glucose concentrations. The results showed that the expression of 3 genes (CLCN7, LIPA, and RRAGD) showed no change at the highest concentration of glucose whereas the other genes had increased expression at all concentrations of glucose. This suggests that for some of the genes, the reduction in expression is not affected by an increased level of sorbitol, while other genes may be influenced by sorbitol accumulation. The role that sorbitol may elicit on the regulation of expression of some of the genes reduced by exposure to elevated glucose concentrations will require further studies. Since sorbitol accumulation is rapid, its effect may be confined to the few genes that were reduced following only one passage of exposure to elevated glucose concentrations. The hRTE cells used in the present study routinely undergo 10 serial passages before a marked reduction in cell growth is noted to occur. This effectively rules out the possibility that the current results were due to cell senescence. The extended length of time necessary for exposure to elevated glucose to reduce the expression of the lysosomal and mTOR genes would be most consistent with a role for non-enzymatic glycosylation [48], although this is not known at the present time.

## Conclusions

In this study we show that exposure of human proximal tubular cells to elevated levels of glucose decreases the expression of lysosomal genes as well as mTOR and its associated genes. Although the mechanism by which glucose causes this decrease is not known, our results suggest that high levels of glucose could decrease the expression of mTOR causing the cells to enter an anabolic state with subsequent downregulation of lysosomal genes.

## Supporting information

S1 Fig Expression of lysosomal markers in cultures of glucose treated human proximal tubular cells. Human proximal tubule cells treated with 5.5mM, 7.5mM, 11mM and 16mM glucose concentrations after first, second and third serial passages.
(TIF)

S2 Fig Expression of mTOR and its associated genes in cultures of glucose treated human proximal tubular cells. Human proximal tubule cells treated with 5.5mM, 7.5mM, 11mM and 16mM glucose concentrations after first, second and third serial passages.
(TIF)

S1 Raw images (TIF)

S1 Table Summary of data processing results. (XLSX)

S2 Table Correlation matrix showing Pearson correlation coefficients between expression profiles of all samples. (XLSX)

S3 Table Results of post hoc tests with ANOVA to explore differences between means. (XLSX)

S4 Table Functional annotation of differentially expressed genes. (XLSX)

S5 Table Subset of genes from S4 Table with low expression at the highest glucose concentration. (XLSX)

S6 Table Subset of genes from S4 Table with high expression at the highest glucose concentration. (CSV)

S7 Table Functional annotation of differentially expressed genes with high expression in the highest glucose concentration. (XLSX)

S8 Table Functional annotation of differentially expressed genes with low expression in highest glucose concentration. (XLSX)

S9 Table List of antibodies used in Western blot and immunofluorescence analysis. (DOCX)

## References

[1] Chevalier RL. The proximal tubule is the primary target of injury and progression of kidney disease: role of the glomerulotubular junction. Am J Physiol Renal Physiol. 2016 Jul 1;311(1): F145–61. 10.1152/ajprenal.00164.2016 27194714 PMC4967168

[2] Gewin LS. Renal fibrosis: Primacy of the proximal tubule. Matrix Biol. 2018 Aug;68–69:248–262. 10.1016/j.matbio.2018.02.006 29425694 PMC6015527

[3] Gilbert RE. Proximal Tubulopathy: Prime Mover and Key Therapeutic Target in Diabetic Kidney Disease. Diabetes. 2017 4;66(4):791–800. 10.2337/db16-0796 28325740

[4] Vallon V. The proximal tubule in the pathophysiology of the diabetic kidney. Am J Physiol Regul Integr Comp Physiol. 2011 5;300(5): R1009–22. 10.1152/ajpregu.00809.2010 21228342 PMC3094037

[5] Zeni L, Norden AGW, Cancarini G, Unwin RJ. A more tubulocentric view of diabetic kidney disease. J Nephrol. 2017 Dec;30(6):701–717. 10.1007/s40620-017-0423-9 Epub 2017 Aug 24. Review. 28840540 PMC5698396

[6] Shrestha S, Garrett SH, Sens DA, Zhou XD, Guyer R, Somji S. Characterization and determination of cadmium resistance of CD133+/CD24+ and CD133-/CD24+ cells isolated from the immortalized human proximal tubule cell line, RPTEC/TERT1. Toxicol Appl Pharmacol. 2019 Jul 15;375:5–16. 10.1016/j.taap.2019.05.007 31078587 PMC6766375

[7] Shrestha S, Somji S, Sens DA, Slusser-Nore A, Patel DH, Savage E, et al. Human renal tubular cells contain CD24/CD133 progenitor cell populations: Implications for tubular regeneration after toxicant induced damage using cadmium as a model. Toxicol Appl Pharmacol. 2017 Sep 15;331:116–129. 10.1016/j.taap.2017.05.038 28587817 PMC5649361

[8] Bussolati B, Bruno S, Grange C, Buttiglieri S, Deregibus MC, Cantino D, et al. Isolation of Renal Progenitor Cells from Adult Human Kidney. Am J. Pathol., 2005 Feb;166(2):545–55. 10.1016/S0002-9440(10)62276-6 15681837 PMC1602314

[9] Lindgren D, Boström AK, Nilsson K, Hansson J, Sjölund J, Möller C, et al. Isolation and characterization of progenitor-like cells from human renal proximal tubules. Am J Pathol. 2011 Feb;178(2):828–37. 10.1016/j.ajpath.2010.10.026 21281815 PMC3070548

[10] Romagnani P, Lasagni L, Remuzzi G. Renal progenitors: an evolutionary conserved strategy for kidney regeneration. Nat Rev Nephrol. 2013 Mar;9(3):137–46. 10.1038/nrneph.2012.290 23338209

[11] Romagnani P, Remuzzi G. CD133+ renal stem cells always co-express CD24 in adult human kidney tissue. Stem Cell Res. 2014 May;12(3):828–9. 10.1016/j.scr.2013.12.011 24467938

[12] Smeets B, Boor P, Dijkman H, Sharma SV, Jirak P, Mooren F, et al. Proximal tubular cells contain a phenotypically distinct, scattered cell population involved in tubular regeneration. J Pathol. 2013 Apr;229(5):645–59. 10.1002/path.4125 23124355 PMC3951144

[13] Hazen-Martin DJ, Sens MA, Detrisac CJ, Blackburn JG, Sens DA. Elevated glucose alters paracellular transport of cultured human proximal tubule cells. Kidney Int. 1989 Jan;35(1):31–9. 10.1038/ki.1989.5 2709660

[14] Bylander JE, Sens DA. Elicitation of sorbitol accumulation in cultured human proximal tubule cells by elevated glucose concentrations. Diabetes. 1990 Aug;39(8):949–54. 10.2337/diab.39.8.949 2115481

[15] Todd JH, Sens MA, Hazen-Martin DJ, Bylander JE, Smyth BJ, Sens DA. Variation in the electrical properties of cultured human proximal tubule cells. In Vitro Cell Dev Biol Anim. 1993 May;29A(5):371–8. 10.1007/BF02633984 8390973

[16] Detrisac CJ, Sens MA, Garvin AJ, Spicer SS, Sens DA. Tissue culture of human kidney epithelial cells of proximal tubule origin. Kidney Int. 1984 Feb;25(2):383–90. 10.1038/ki.1984.28 6727133

[17] Kim D, Garrett SH, Sens MA, Somji S, Sens DA. Metallothionein isoform 3 and proximal tubule vectorial active transport. Kidney Int. 2002 Feb;61(2):464–72. 10.1046/j.1523-1755.2002.00153.x 11849386

[18] Garrett SH, Somji S, Todd JH, Sens DA. Exposure of human proximal tubule cells to Cd2+, Zn2+, and Cu2+ induces metallothionein protein accumulation but not metallothionein isoform 2 mRNA. Environ Health Perspect. 1998 Sep;106(9):587–95. 10.1289/ehp.98106587 9721259 PMC1533161

[19] Bathula CS, Garrett SH, Zhou XD, Sens MA, Sens DA, Somji S. Cadmium, vectorial active transport, and MT-3-dependent regulation of cadherin expression in human proximal tubular cells. Toxicol Sci. 2008 4;102(2):310–8. 10.1093/toxsci/kfn004 18182399

[20] Slusser A, Bathula CS, Sens DA, Somji S, Sens MA, Zhou XD, et al. Cadherin expression, vectorial active transport, and metallothionein isoform 3 mediated EMT/MET responses in cultured primary and immortalized human proximal tubule cells. PLoS One. 2015;10(3):e0120132. 10.1371/journal.pone.0120132 25803827 PMC4372585

[21] Dunning MJ, Smith ML, Ritchie ME, Tavaré S. beadarray: R classes and methods for Illumina bead-based data. Bioinformatics. 2007 Aug 15;23(16):2183–4. 10.1093/bioinformatics/btm311 17586828

[22] Myers J.L., Well A., and Lorch R.F., *Research design and statistical analysis*. 3rd ed. 2010, New York: Routledge. 809 p.

[23] *Pearson’s Correlation Coefficient*, in *Encyclopedia of Public Health*, Kirch W., Editor. 2008, Springer Netherlands: Dordrecht. pp 1090–1091.

[24] Kim HY. Analysis of variance (ANOVA) comparing means of more than two groups. Restor Dent Endod. 2014 Feb;39(1):74–7. 10.5395/rde.2014.39.1.74 24516834 PMC3916511

[25] Peto R. Current misconception 3: that subgroup-specific trial mortality results often provide a good basis for individualising patient care. Br J Cancer. 2011 Mar 29;104(7):1057–8. 10.1038/bjc.2011.79 21448174 PMC3068511

[26] Haynes W., *Benjamini–Hochberg Method*, in *Encyclopedia of Systems Biology*, Dubitzky W., et al., Editors. 2013, Springer New York: New York, NY. p. 78–78.

[27] Kanehisa M, Goto S, Sato Y, Furumichi M, Tanabe M. KEGG for integration and interpretation of large-scale molecular data sets. Nucleic Acids Res. 2012 1;40 (Database issue):D109–14. 10.1093/nar/gkr988 22080510 PMC3245020

[28] DennisGJr, ShermanBT, HosackDA, YangJ, GaoW, LaneHC, et al. DAVID: Database for Annotation, Visualization, and Integrated Discovery. Genome Biol. 2003;4(5):P3. Epub 2003 Apr 3. 12734009

[29] Stelzer G, Rosen N, Plaschkes I, Zimmerman S, Twik M, Fishilevich S, et al. The GeneCards Suite: From Gene Data Mining to Disease Genome Sequence Analyses. Curr Protoc Bioinformatics. 2016 Jun 20;54:1.30.1–1.30.33. 10.1002/cpbi.5 27322403

[30] Huang da W, Sherman BT, Lempicki RA. Systematic and integrative analysis of large gene lists using DAVID bioinformatics resources. Nat Protoc. 2009;4(1):44–57. 10.1038/nprot.2008.211 19131956

[31] Huang da W, Sherman BT, Lempicki RA. Bioinformatics enrichment tools: paths toward the comprehensive functional analysis of large gene lists. Nucleic Acids Res. 2009 Jan;37(1):1–13. 10.1093/nar/gkn923 19033363 PMC2615629

[32] Fabregat A, Jupe S, Matthews L, Sidiropoulos K, Gillespie M, Garapati P, et al. The Reactome Pathway Knowledgebase. Nucleic Acids Res. 2018 Jan 4;46(D1):D649–D655. 10.1093/nar/gkx1132 29145629 PMC5753187

[33] Rong Y, McPhee CK, Deng S, Huang L, Chen L, Liu M, et al. Spinster is required for autophagic lysosome reformation and mTOR reactivation following starvation. Proc Natl Acad Sci U S A. 2011 May 10;108(19):7826–31. 10.1073/pnas.1013800108 21518918 PMC3093520

[34] Vallon V, Platt KA, Cunard R, Schroth J, Whaley J, Thomson SC, et al. SGLT2 mediates glucose reabsorption in the early proximal tubule. J Am Soc Nephrol. 2011 Jan;22(1):104–12. 10.1681/ASN.2010030246 20616166 PMC3014039

[35] Song P, Huang W, Onishi A, Patel R, Kim YC, van Ginkel C, et al. Knockout of Na+-glucose cotransporter SGLT1 mitigates diabetes-induced upregulation of nitric oxide synthase NOS1 in the macula densa and glomerular hyperfiltration. Am J Physiol Renal Physiol. 2019 Jul 1;317(1):F207–F217. 10.1152/ajprenal.00120.2019 31091127 PMC6692722

[36] Lawrence RE, Zoncu R. The lysosome as a cellular centre for signalling, metabolism and quality control. Nat Cell Biol. 2019 Feb;21(2):133–142. 10.1038/s41556-018-0244-7 30602725

[37] Rabanal-Ruiz Y, Korolchuk VI. mTORC1 and Nutrient Homeostasis: The Central Role of the Lysosome. Int J Mol Sci. 2018 Mar 12;19(3). 10.3390/ijms19030818 29534520 PMC5877679

[38] Settembre C, Ballabio A. Lysosomal adaptation: how the lysosome responds to external cues. Cold Spring Harb Perspect Biol. 2014 May 5;6(6). 10.1101/cshperspect.a016907 24799353 PMC4031961

[39] Surendran K, Vitiello SP, Pearce DA. Lysosome dysfunction in the pathogenesis of kidney diseases. Pediatr Nephrol. 2014 Dec;29(12):2253–61. 10.1007/s00467-013-2652-z 24217784 PMC4018427

[40] Wang W, He Q, Yan W, Sun J, Chen Z, Liu Z, et al. High glucose enhances the metastatic potential of tongue squamous cell carcinoma via the PKM2 pathway. Oncotarget. 2017 Dec 19;8(67):111770–111779. 10.18632/oncotarget.22907 29340090 PMC5762358

[41] Seebacher NA, Lane DJ, Jansson PJ, Richardson DR. Glucose Modulation Induces Lysosome Formation and Increases Lysosomotropic Drug Sequestration via the P-Glycoprotein Drug Transporter. J Biol Chem. 2016 Feb 19;291(8):3796–820. 10.1074/jbc.M115.682450 26601947 PMC4759162

[42] Palmieri M, Impey S, Kang H, di Ronza A, Pelz C, Sardiello M, et al. Characterization of the CLEAR network reveals an integrated control of cellular clearance pathways. Hum Mol Genet. 2011 Oct 1;20(19):3852–66. 10.1093/hmg/ddr306 21752829

[43] Sardiello M, Palmieri M, di Ronza A, Medina DL, Valenza M, Gennarino VA, et al. A gene network regulating lysosomal biogenesis and function. Science. 2009 Jul 24;325(5939):473–7. 10.1126/science.1174447 19556463

[44] Lim CY, Zoncu R. The lysosome as a command-and-control center for cellular metabolism. J Cell Biol. 2016 Sep 12;214(6):653–64. 10.1083/jcb.201607005 27621362 PMC5021098

[45] Efeyan A, Zoncu R, Chang S, Gumper I, Snitkin H, Wolfson RL, et al. Regulation of mTORC1 by the Rag GTPases is necessary for neonatal autophagy and survival. Nature. 2013 Jan 31;493(7434):679–83. 10.1038/nature11745 23263183 PMC4000705

[46] Bendavit G, Aboulkassim T, Hilmi K, Shah S, Batist G. Nrf2 Transcription Factor Can Directly Regulate mTOR: Linking cytoprotective gene expression to a major metabolic regulator that generates redox activity. J Biol Chem. 2016 Dec 2;291(49):25476–25488. 10.1074/jbc.M116.760249 27784786 PMC5207248

[47] de Haan JB. Nrf2 activators as attractive therapeutics for diabetic nephropathy. Diabetes. 2011 Nov;60(11):2683–4. 10.2337/db11-1072 22025774 PMC3198074

[48] Rowan S, Bejarano E, Taylor A. Mechanistic targeting of advanced glycation end-products in age-related diseases. Biochim Biophys Acta Mol Basis Dis. 2018; 1864(12): 3631–3643. 10.1016/j.bbadis.2018.08.036 30279139 PMC6822271

