# Supplementary figures and images for: Elevated glucose represses lysosomal and mTOR-related genes in renal epithelial cells composed of progenitor CD133+ cells
Source: PLoS One. 2021 Mar 25;16(3):e0248241. doi: 10.1371/journal.pone.0248241 (PMC7993790; doi:10.1371/journal.pone.0248241)

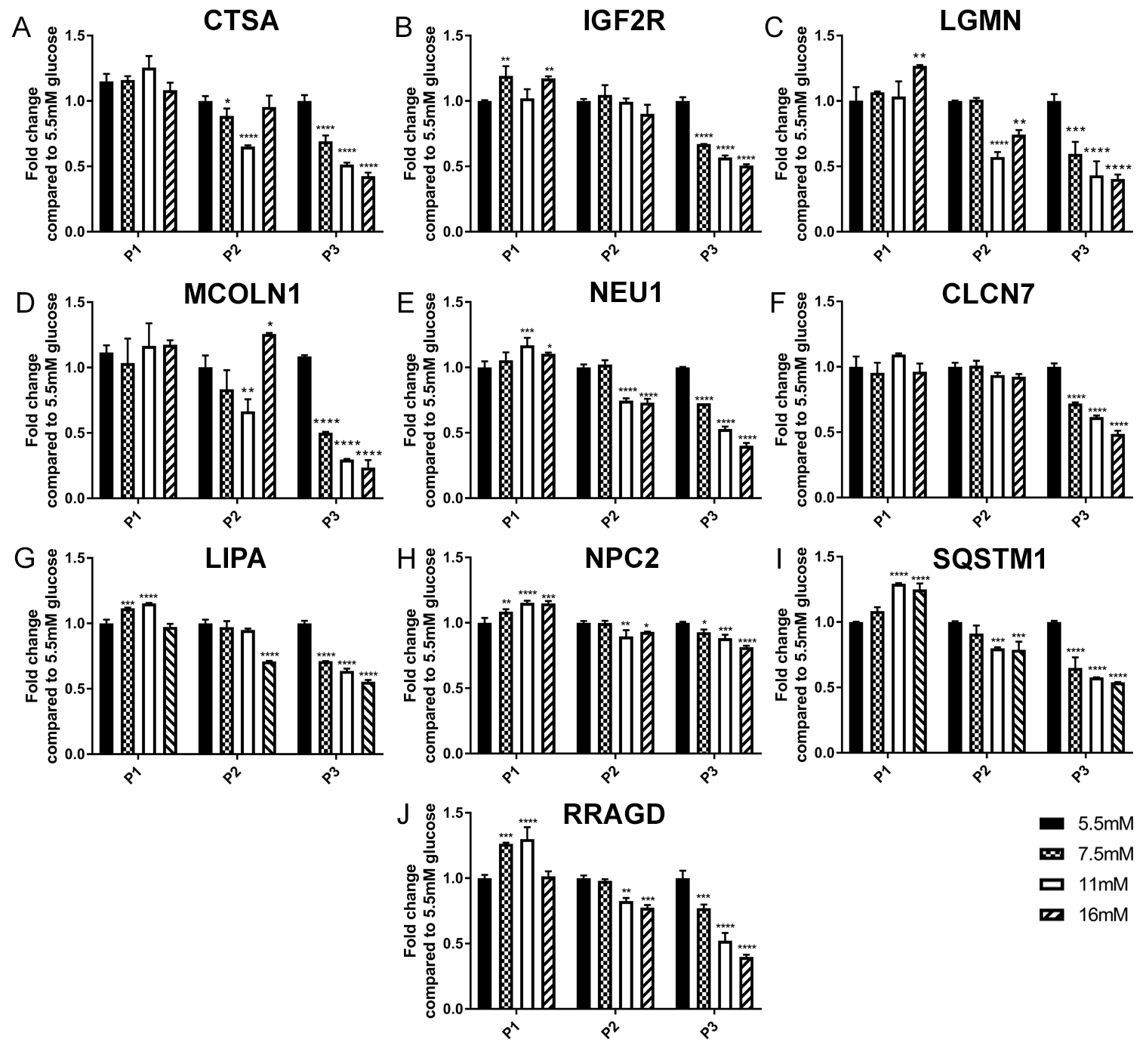

Supplement: S1 Fig — Human proximal tubule cells treated with 5.5mM, 7.5mM, 11mM and 16mM glucose concentrations after first, second and third serial passages. (TIF) [file pone.0248241.s001.tif]

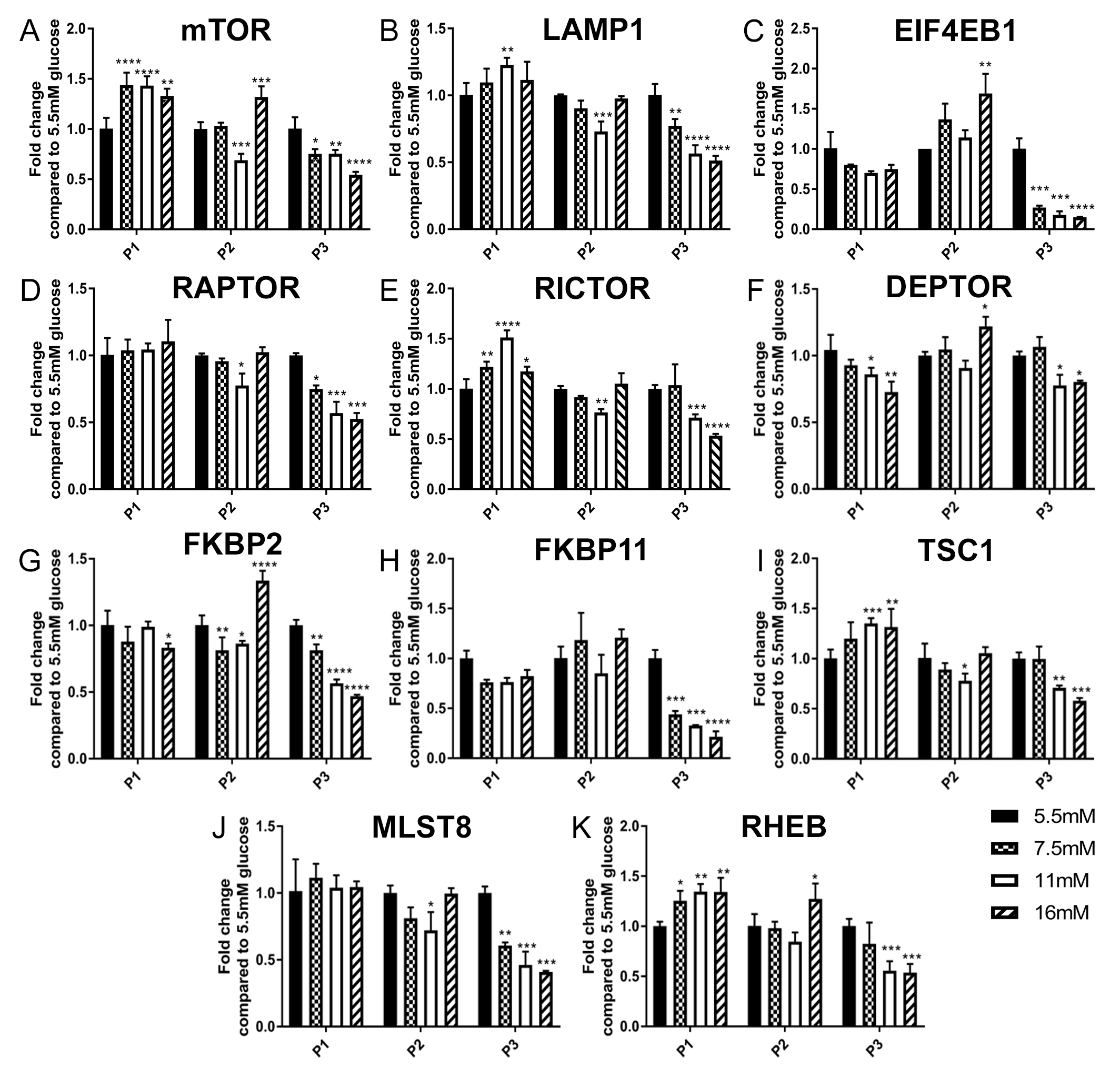

Supplement: S2 Fig — Human proximal tubule cells treated with 5.5mM, 7.5mM, 11mM and 16mM glucose concentrations after first, second and third serial passages. (TIF) [file pone.0248241.s002.tif]

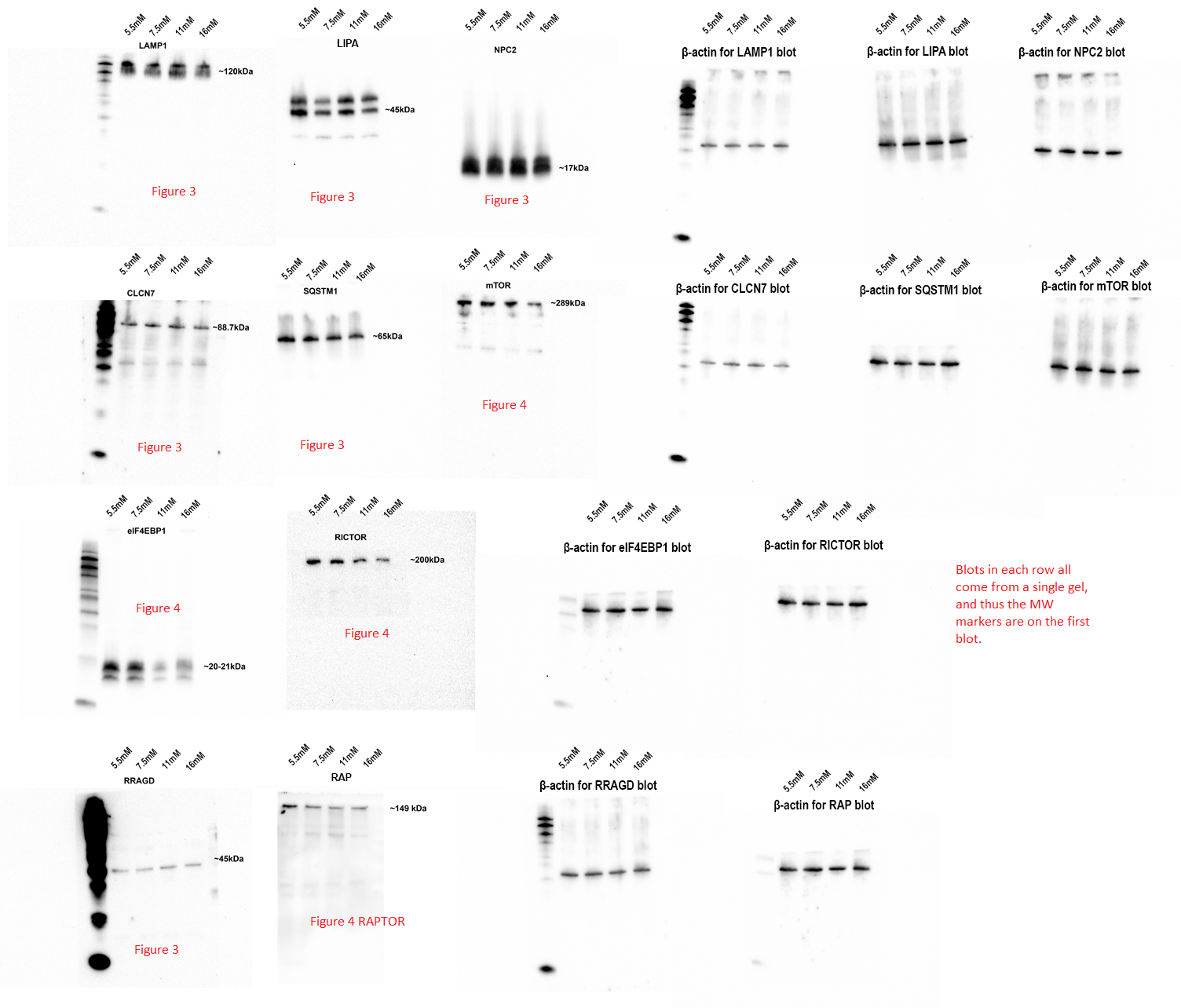

Supplement: S1 Raw images — (TIF) [file pone.0248241.s003.tif]
