# Supplementary material for: Elevated glucose represses lysosomal and mTOR-related genes in renal epithelial cells composed of progenitor CD133+ cells
Source: PLoS One. 2021 Mar 25;16(3):e0248241. doi: 10.1371/journal.pone.0248241 (PMC7993790; doi:10.1371/journal.pone.0248241)
Supplement: S9 Table — (DOCX) [file pone.0248241.s012.docx]

| Antigen | Abbreviation | Source | Catalog # | Dilution | |
| --- | --- | --- | --- | --- | --- |
|  |  |  |  | WB | IF |
| H(+)/Cl(-) exchange transporter 7 | CLCN7 | Novus Biologicals | NBP2-30021 | 1:1000 | 1:20 |
| Lysosomal acid lipase/cholesteryl ester hydrolase | LIPA | Novus Biologicals | NBP1-54155 | 1:1500 |  |
| Anti-Niemann Pick C2 | NPC2 | Abcam | ab218192 | 1:2000 | 1:1000 |
| Eukaryotic translation initiation factor 4E-binding protein 1 | eIF4EBP1 | Abcam | ab2606 | 1:2000 | - |
| Ras-related GTP-binding protein C | RRAGC | Sigma Life Science | HPA055489 | - | 1:120 |
| Ras-related GTP-binding protein D | RRAGD | Thermofisher/  Invitrogen | PA5-67003 | 1:250 | 1:25 |
| Sequestosome-1 | SQSTM1 | Abcam | ab56416 | 1:2000 | 1:125 |
| Mammalian Target of Rapamycin | mTOR | Cell Signaling | 7C10 | 1:1000 | 1:400 |
| Lysosome-associated membrane glycoprotein 1 | LAMP1 | SantaCruz Biotechnology | sc-20011 | 1:1500 | 1:100 |
| Regulatory-associated protein of mTOR | RPTOR | SantaCruz Biotechnology | sc-81537 | 1:500 | - |
| Rapamycin-insensitive companion of mTOR | RICTOR | SantaCruz Biotechnology | sc-271081 | 1:500 | - |
| Beta-actin | β-actin | abcam | ab8227 | 1:1000 | - |

S9 Table. List of antibodies used in Western Blot (WB) and immunofluorescence (IF) microscopy:
